# Chronic ethanol alters network activity and endocannabinoid signaling in the prefrontal cortex

**DOI:** 10.3389/fnint.2014.00058

**Published:** 2014-07-18

**Authors:** Matthew J. Pava, John J. Woodward

**Affiliations:** Department of Neuroscience, Medical University of South CarolinaCharleston, SC, USA

**Keywords:** up-states, CB1, WIN, electrophysiology, GABA, glutamate

## Abstract

Chronic use of alcohol is associated with structural and functional alterations in brain areas that subserve cognitive processes. Of particular importance is the prefrontal cortex (PFC) that is involved in higher order behaviors such as decision making, risk assessment and judgment. Understanding the mechanisms that underlie alcohol's effects on PFC function is important for developing strategies to overcome the cognitive deficits that may predispose individuals to relapse. Our previous studies showed that acutely applied ethanol inhibits network activity in slices of prefrontal cortex and that exogenous and endogenous cannabinoids modulate up-state dynamics. In the present study, we examined the effects of repeated alcohol exposure on cannabinoid regulation of up-states in slice cultures of the prefrontal cortex. Compared to controls, up-state duration, but not amplitude was enhanced when measured 4 days after a 10 day ethanol exposure (44 mM ethanol; equivalent to 0.2% blood ethanol). Administration of the CB1 agonist WIN 55,212-2 enhanced the amplitude of up-states in control cultures but not in those treated previously with ethanol. This lack of effect occurred in the absence of any noticeable change in CB1 receptor protein expression. Chronic ethanol treatment and withdrawal also blunted WIN's inhibition of electrically evoked GABA IPSCs in layer II/III pyramidal neurons but not those in layer V/VI. WIN inhibited the amplitude of spontaneous GABA IPSCs in both layers and the magnitude of this effect was not altered by ethanol treatment. However, in layer V/VI neurons, WIN's effect on sIPSC frequency was greater in ethanol treated cultures. WIN also inhibited electrically evoked NMDA EPSCs in both layer II/III and V/VI neurons but this action was unaffected by ethanol treatment and withdrawal. Overall, these results suggest that ethanol's down-regulation of cannabinoid signaling results in altered network activity in the prefrontal cortex.

## Introduction

Addictive disorders are associated with cognitive deficits that often persist into abstinence and may arise from dysfunction of the prefrontal cortex (PFC; Koob and Volkow, 2010; Lundqvist, 2010). Because of the PFC's role in controlling drug-seeking behaviors, the persistent disruption of PFC function by chronic drug or alcohol exposure may contribute to relapse following periods of withdrawal and abstinence (Lasseter et al., 2010). Long-term heavy alcohol use such as that associated with alcohol dependence produces structural changes to the PFC (Pfefferbaum et al., 1992; Chanraud et al., 2007), but it is likely that changes in physiological processes that result in reduced or aberrant cortical function presage structural abnormalities. In support of this, studies with human alcohol dependent patients have reported correlated reductions in behavioral and physiological indices of frontal cortical function including reduced power of the delta frequency in EEG recordings (Kamarajan et al., 2004; Chen et al., 2007).

In addition to cognitive deficits that can persist well into abstinence, alcohol dependent patients often also experience disruptions in sleep (Junghanns et al., 2009), and alcohol dependent mice exhibit reduced sleep efficiency and time spent in non-REM sleep (Veatch, 2006). One form of cortical network activity that contributes to slow frequencies in the delta band (0–1 Hz) and predominates in deep-stage slow-wave sleep is the slow-oscillation (Amzica and Steriade, 1998). This EEG rhythm arises from the bi-stable membrane potential of cortical neurons (Steriade et al., 1993a,b,c) that spontaneously oscillates between a quiescent, hyperpolarized “down-state” and periods of depolarization called “up-states” that are crowned with robust synaptic activity and action potentials. The shift between up- and down-states is a network event because the transitions into and out of these phases are synchronized across large populations of neurons in the cortex (Sanchez-Vives and McCormick, 2000; Seamans et al., 2003; Woodward and Pava, 2009, 2012). This large-scale network activation is generated through the effects of mixed glutamatergic and GABAergic synaptic transmission with glutamate-activated currents serving as a depolarizing force moderated by GABA-mediated conductance that provides a clamp near the Cl^−^ reversal potential (≈ −55 mV). As a result, up-states are dependent upon NMDA, AMPA, and GABA_A_ currents, and up-state parameters are significantly altered by inhibition of these receptors with selective antagonists (Seamans et al., 2003) or abused drugs including ethanol (EtOH; Tu et al., 2007).

Although glutamate and GABA are essential for maintaining membrane bi-stability and the slow oscillation, neuromodulators that are associated with different aspects of cognitive function can modify the expression of up-states. For instance, the endocannabinoid (EC) system regulates up-states and non-REM sleep (Pava et al., 2014), and numerous studies have reported significant alteration in the function of the EC system following chronic EtOH treatment (CET; Glaser et al., 2003). Specifically, repeated exposure of mice to EtOH produces widespread alterations in behavioral and physiological responses to a CB1 agonist (Pava et al., 2012) and other studies report that chronic EtOH treatment (CET) reduces CB1 expression and function in whole brain (Basavarajappa et al., 1998; Basavarajappa and Hungund, 1999), hippocampus (Mitrirattanakul et al., 2007), and cortex (Vinod et al., 2006). Additional studies with alcohol preferring rats (Hansson et al., 2007) or mice with a genetic knockout of fatty acid amide hydrolase (FAAH; Vinod et al., 2008) indicate that the propensity to consume EtOH is correlated with high levels of anandamide (AEA) and reduced expression of CB1 in the PFC. These findings, combined with those discussed above, suggest that altered EC signaling in the cortex may be one factor that contributes to the frontal deficits often observed in alcohol dependent individuals.

Given the modulatory effects of the EC system on up-states and the perturbations in EC function following CET, the present study examined whether CET disrupts EC modulation of neocortical up-states. Using a novel PFC slice culture system that generates spontaneous episodes of membrane bi-stability, we show that the duration of up-states is increased following withdrawal from CET. In addition, the ability of a CB1 agonist to alter up-state parameters is reduced in CET exposed cultures and this appears to be due to a reduction in CB1 function that is specific to GABAergic synapses on layer II/III PNs. These findings demonstrate that CET alters normal cortical network activity via reduced signaling through CB1 receptors.

## Methods

### Mice

All mouse pups used in this study were obtained from breeding colonies maintained at the Medical University of South Carolina. Breeding pairs of C57BL6/J mice were originally obtained from the Jackson Laboratories (Bar Harbor, ME). Housing and treatment of all animals used in this study conformed to the Guide for the Care and Use of Laboratory Animals (National Research Council Committee for the Update of the Guide for the Care and Use of Laboratory Animals Institute for Laboratory Animal Research and National Academies Press, 2011).

### Cortical organotypic cultures

Slice cultures of neonatal mouse cerebral cortex were prepared as previously described with some modifications (Tu et al., 2007). Postnatal day 1–5 mouse pups were deeply anesthetized by placing them in an ice water bath for at least 2 min prior to decapitation. Brains were extracted and immediately submerged in ice-cold sterile-filtered HEPES-buffered, sucrose dissection solution containing (in mM): 200 sucrose, 1.9 KCl, 6 MgCl_2_, 0.5 CaCl_2_, 10 glucose, 0.4 ascorbic acid, 25 HEPES, pH 7.3. Coronal sections (400 μm) containing the anterior cingulate (ACC), prelimbic and infralimbic regions of the prefrontal cortex were prepared on a vibrating microtome under sterile conditions in a biosafety hood equipped with laminar flow. Slices representing the right and left hemispheres of each section were transferred to Millicell-CM 0.4 μm biopore membrane inserts in six-well culture plates containing 1 mL pre-incubated high-serum media. This media contained 50% BME, 25% Earl's balanced salt solution, 25% heat-inactivated horse serum (HIHS), 5.9 mg/mL glucose, 25 mM HEPES, 100 μg/mL streptomycin, and 0.235 mM Glutamax. Slices were arranged on the membranes so that their dorsal surfaces were aligned and their medial surfaces were touching.

Cultures were maintained in an incubator at 37°C and a 5% CO_2_ atmosphere with partial media exchanges every 2–3 days. Beginning at 3 days *in vitro* (DIV), high-serum media was replaced with media containing 5% HIHS. At 14 DIV, culture media was supplemented with 20 μM 5-fluoro-2-deoxyuridine to prevent glial overgrowth. All recordings from cultures were made after 14 DIV to allow recovery from slicing and for the cortical network to mature.

### Chronic EtOH treatment (CET)

Cultures were exposed to CET (44 mM) or control (CNTRL; no EtOH) conditions over 10 days in sealed vapor chambers as previously described (Chandler et al., 1993; Carpenter-Hyland et al., 2004). At 14 DIV culture media was replaced with EtOH-containing media, and culture plates were placed into Tupperware chambers with an open plastic reservoir filled with 250 mL of 1% SDS/44 mM EtOH solution. The chambers were gassed with a mixture of 5% CO2 balanced with air before being sealed and placed inside a culture incubator maintained at 37°C. CNTRL cultures were treated identically to CET cultures with the exception that neither the culture media nor the reservoir contained EtOH. On the tenth day of treatment, CET and CNTRL cultures were removed from the vapor chambers for a full media exchange with non-EtOH containing media before being placed back into the incubator. For immunoblots, some cultures were scraped immediately into microcentrifuge tubes upon removal from the vapor chambers to prevent them from undergoing withdrawal. All other cultures underwent 4 days of withdrawal prior to use in immunoblots or physiological experiments.

### Whole-cell electrophysiology

On the day of recording, cultures were removed from the incubator, and the membrane immediately surrounding the culture was cut from the rest of the insert while taking care not to damage the tissue. The culture was then submerged in a recording chamber perfused at 2 mL/min with ACSF containing (in mM): 125 NaCl, 2.5 KCl, 1.25 NaH_2_PO_4_, 1.3 MgCl_2_, 2.0 CaCl_2_, 0.4 ascorbic acid, 10 glucose, 25 NaHCO_3_, 0.05% bovine serum albumin and continuously bubbled with carbogen gas (95% O_2_/5% CO_2_). Bath temperature was maintained at 32.0 ± 0.5°C using a heated recording chamber and an in-line flow-through heater controlled by a thermistor-coupled TC-342B temperature controller (Warner Instruments, Hampden, CT). For current-clamp experiments, patch-pipettes (1.5 × 1.1 mm; 1.8 – 3.5 MΩ) were filled with internal recording solution containing (in mM): 130 K-gluconate, 10 KCl, 2 MgCl_2_, 0.1 EGTA, 10 HEPES, 2 NaATP, 0.3 NaGTP, pH 7.3. For voltage-clamp recordings, patch-pipettes were filled with a solution containing (in mM): 140 CsCl, 2 MgCl_2_, 0.1 EGTA, 10 HEPES, 2 NaATP, 0.3 NaGTP, 5 QX-314, pH 7.3. Whole-cell patch-clamp recordings were made from visually identified pyramidal neurons (PN) in the region of cultured cortex corresponding to the ACC. Neurons were imaged using a Zeiss FS2 microscope (Oberkochen, Germany) equipped with an infrared video camera and Dodt gradient contrast optics. For all recordings, gigaohm seals were obtained in voltage-clamp mode using an Axoclamp 700A amplifier (Molecular Devices, Sunnyvale, CA). For current-clamp experiments, the amplifier mode was switched following breakthrough. Pipette access resistance (5–25 MΩ) was monitored throughout experiments and cells showing a significant deviation in access resistance (>25%) were not used for analysis. Square-wave electrical stimuli (0.1 ms) used to evoke up-states or post-synaptic currents (PSCs) were generated by a stimulus isolation unit and delivered to the tissue via a concentric bipolar electrode placed in the cortical tissue. For current clamp recordings up-states were evoked with a 750 μA pulse delivered at 0.033 Hz to the lateral aspect of the cortex, distal to the recording site. For voltage clamp recordings of evoked PSCs, the stimuli (75–200 μA) were delivered to cortical layer II/III proximal to the recording electrode at 0.05 Hz.

GABA_A_-mediated inhibitory PSCs (IPSCs) were recorded from neurons held at −70 mV in ACSF supplemented with (*R*,S)-amino-5-phosphonovaleric acid (DL-APV; 100 μM), 6,7-dinitroquinoxaline-2,3-dione (DNQX; 20 μM), and (2***S***)-3-[[(1***S***)-1-(3,4-Dichlorophenyl)ethyl]amino-2-hydroxypropyl] (phenylmethyl) phosphinic acid (CGP-55845; 1 μM) to block NMDA-, AMPA-, and GABA_B_- mediated responses, respectively. NMDA-mediated excitatory PSCs (EPSCs) were recorded from cells held at +40 mV in ACSF supplemented with picrotoxin (100 μM) and DNQX. Recordings from AMPA-mediated EPSCs are not reported because it was not possible to obtain monosynaptic responses from the organotypic cultures used in this study. This is likely due to the highly ramified synaptic structure of these cultures that allows for the generation of up-states. Data for all experiments were acquired at 10 kHz and filtered at 4 kHz. The amplifier output was digitized by an ITC-16 interface (Instrutech, Port Washington, NY) controlled by AxoGraphX software (AxoGraph Scientific, Sydney, Australia) running on a Macintosh G4 (Apple Inc., Cupertino, CA).

### Western blots

Tissue from cultures was harvested for cell fractionation either immediately following treatment or after a 4 day withdrawal. Prior to scraping, cultures were washed three times to remove excess media in an ice-cold HEPES-buffered sucrose solution containing (in mM): 200 sucrose, 1.9 KCl, 6 MgCl_2_, 0.5 CaCl_2_, 10 glucose, 0.4 ascorbic acid, 25 HEPES, pH 7.3 with KOH.

A crude membrane fraction was prepared and tissue samples were used for western blotting as previously described (Mulholland and Chandler, 2010). After separation on NuPage Novex gels (4–12% Bis-Tris; Invitrogen Corp., Carlsbad, CA) protein was transferred to Immobilon-P PVDF membranes (Millipore Corporation, Billerica, MA). Membranes were blocked in 1% non-fat dry milk plus 5% BSA and incubated at 4°C overnight in a 1:500 dilution of the L15 anti-CB1 primary antibody (kindly provided by Dr. Ken Mackie, Indiana University, Bloomington, IN). A horseradish peroxidase (HRP) conjugated goat anti-rabbit secondary antibody was then added and bands were detected using enhanced chemiluminescence (ECL). The band corresponding to the molecular weight of CB1 (≈52 kDa) was quantified using computer-assisted densitometry with ImageJ v1.41 (National Institutes of Health, USA).

### Statistical analysis

Statistical analysis of data was performed using Prism 4 (GraphPad Software, San Diego, CA). Bonferroni *post-hoc* tests were conducted for all analysis of variance (ANOVA) tests when appropriate. For measures of PSC inhibition by WIN:

$$\begin{array}{l} -1\times \%\text{change}=\%\text{Inhibition}\\ \;=\frac{\text{Baseline PSC}-\text{Post-WIN PSC}}{\text{Baseline PSC}}\times 100\% \end{array}$$

For all analyses α = 0.05.

## Results

### Enhanced up-state duration and decreased sensitivity to WIN following CET

**Figure 1:**
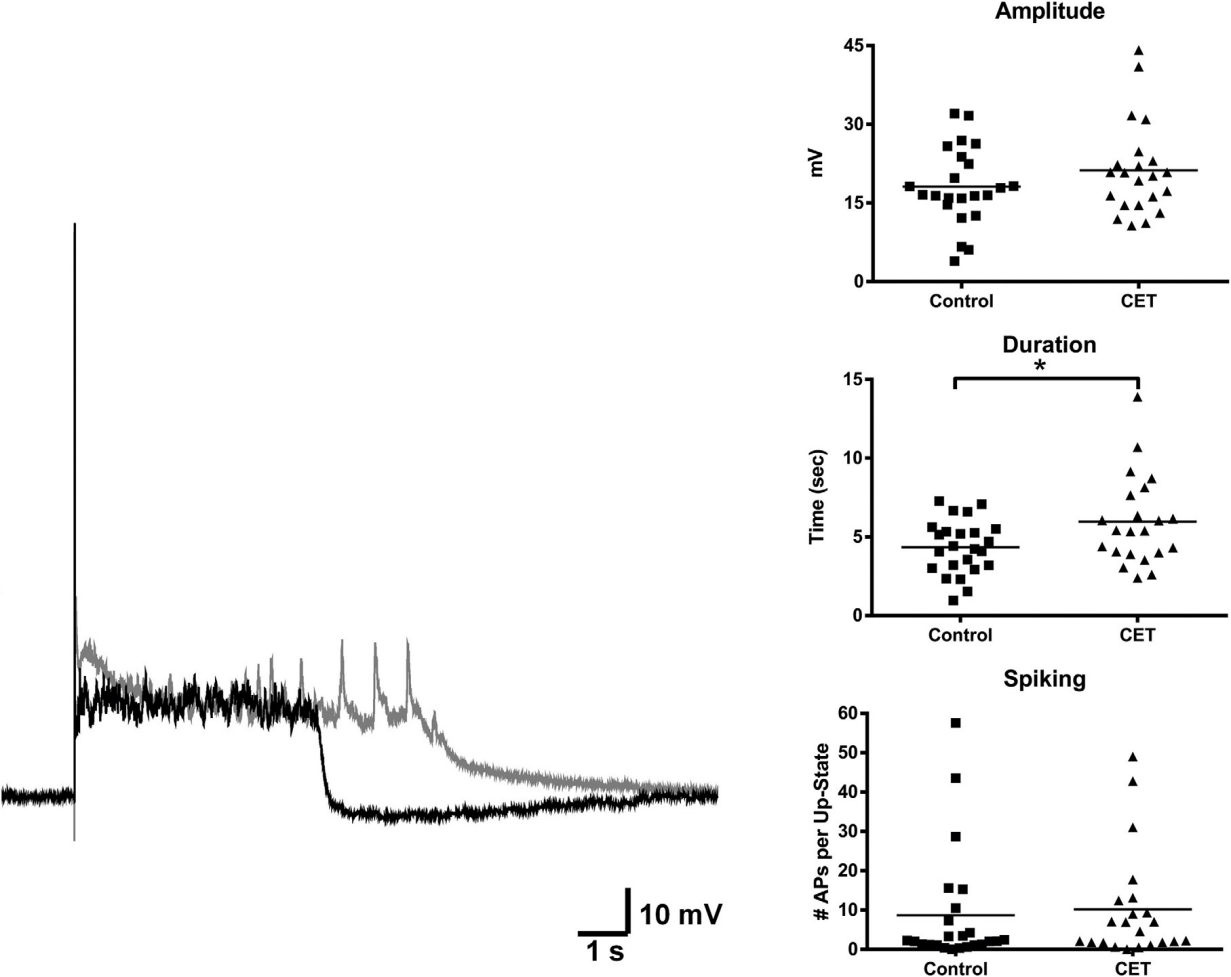
**Up-state duration is increased in cultures after withdrawal from chronic treatment with 44 mM EtOH**. Example traces: black trace is a representative example of an up-state from a control treated culture and gray trace is an example from a chronic EtOH culture. Graphs show summary data of baseline measures of up-state parameters in control and CET culture (*N* = 23–26). Horizontal lines represent group means. Asterisks represent significant group differences (*p* < 0.05).

**Figure 2:**
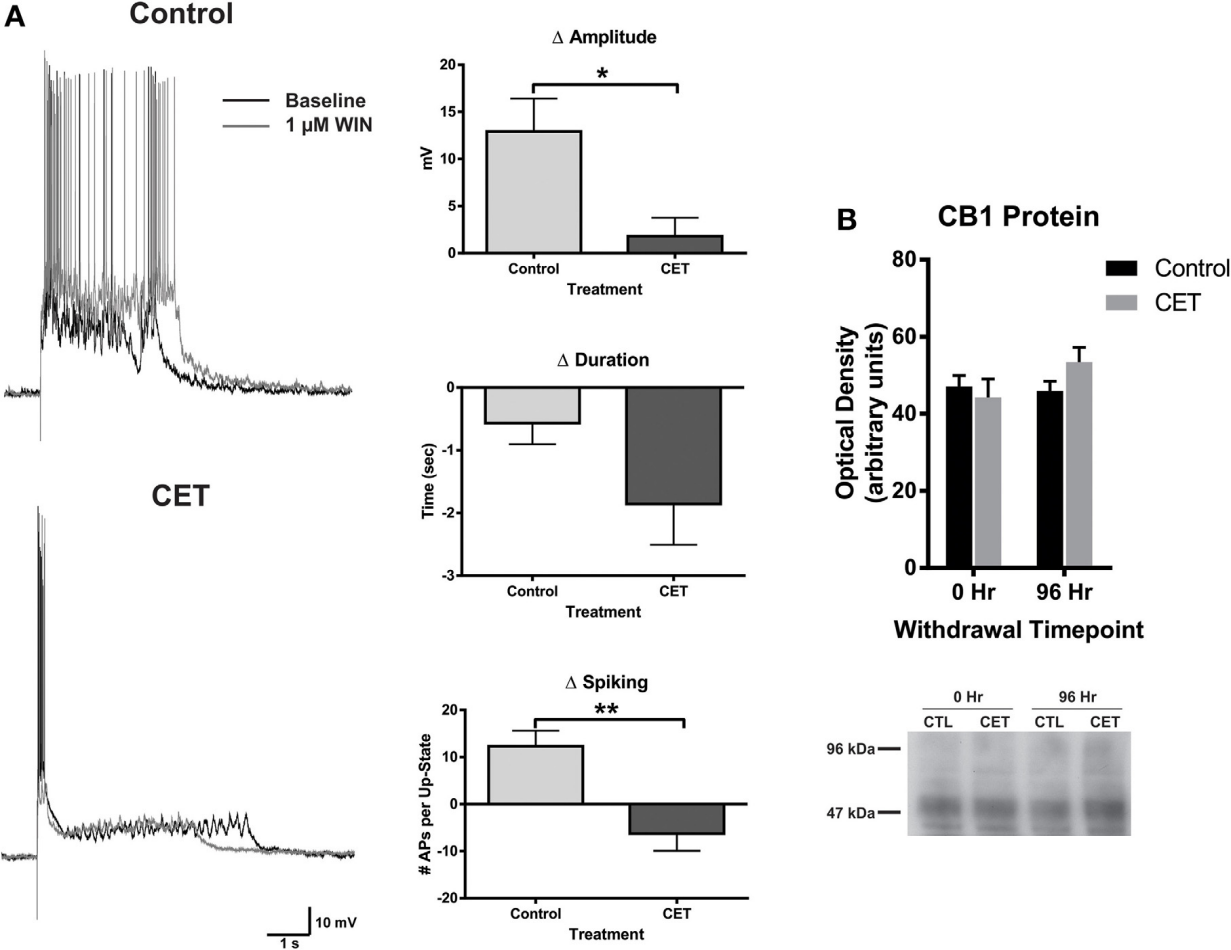
**The effects of WIN are blunted in cultures chronically treated with 44 mM EtOH, without changes in CB1 protein expression**. **(A)** Left: representative traces of up-states recorded from layer V/VI PNs in control and CET cultures under baseline conditions and after application of the CB1 agonist WIN (1 μM). **(A)** Right: summary data from the experiments comparing the WIN-induced change in up-state parameters between control and CET cultures. **(B)** Top: summary data for CB1 immunoblots expressed as optical density of 52 kDa band (*N* = 3–4). Bars represent mean ± s.e.m. Asterisks represent significant group differences: ^*^p < 0.05 and ^**^p < 0.01.

To examine the effects of a 4 day withdrawal from CET on network activity, recordings of evoked up-states were compared between control and EtOH treated slice cultures of mouse PFC (Figure 1). CET cultures recorded after withdrawal had significantly longer up-states compared to controls [*t*-test, *t*_(34)_ = 2.33, *p* = 0.026]. However, neither the number of action potentials during up-states [APs; spiking, *t*_(44)_ = 0.36, *p* = 0.72] nor the amplitude of up-states [*t*_(45)_ = 0.50, *p* = 0.62] differed between treatment groups. A similar enhancement of up-state duration is observed in cultures prepared from CB1 KO mice (Pava et al., 2014), and withdrawal from chronic EtOH administration alters patterns of slow-wave sleep (Veatch, 2006; Mukherjee and Simasko, 2009) and CB1 protein expression in the rodent brain (Basavarajappa et al., 1998; Mitrirattanakul et al., 2007; Pava et al., 2012). In order to determine if CET also altered CB1 function, the effect of the CB1 agonist, WIN 55,212-2 (WIN; 1 μM), on up-state parameters was examined in CET cultures after 4 days of withdrawal (Figure 2A). As reported previously, acute application of WIN increased up-state amplitude and spiking (Pava et al., 2014), but in cultures that had undergone a 4 day recovery from CET, the ability of WIN to increase up-state amplitude [*t*-test, *t*_(13)_ = 2.9, *p* = 0.013] and spiking [*t*_(12)_ = 3.8, p = 0.0026] was significantly blunted compared to control treated cultures. WIN failed to alter up-state duration in either treatment group [*t*_(13)_ = 1.7, p = 0.12] consistent with previous findings. We hypothesized that the down-regulation of CB1 function could arise from reduced CB1 protein and performed western blots for CB1 on crude membrane fractions from CET and control cultures at two time points following ethanol treatment (0 h and 96 h; Figure 2B). Surprisingly, there was not an interaction between withdrawal time and treatment [Two-Way ANOVA, *F*_(1, 9)_ = 2.2, *p* = 0.17], and there was no main effect of CET on CB1 protein content [*F*_(1, 9)_ = 0.44, *p* = 0.52]. These results suggest that withdrawal from CET produces a down-regulation of CB1 function without affecting overall CB1 expression.

### CET produces layer specific changes in WIN-induced depression of GABAergic IPSCs

**Figure 3:**
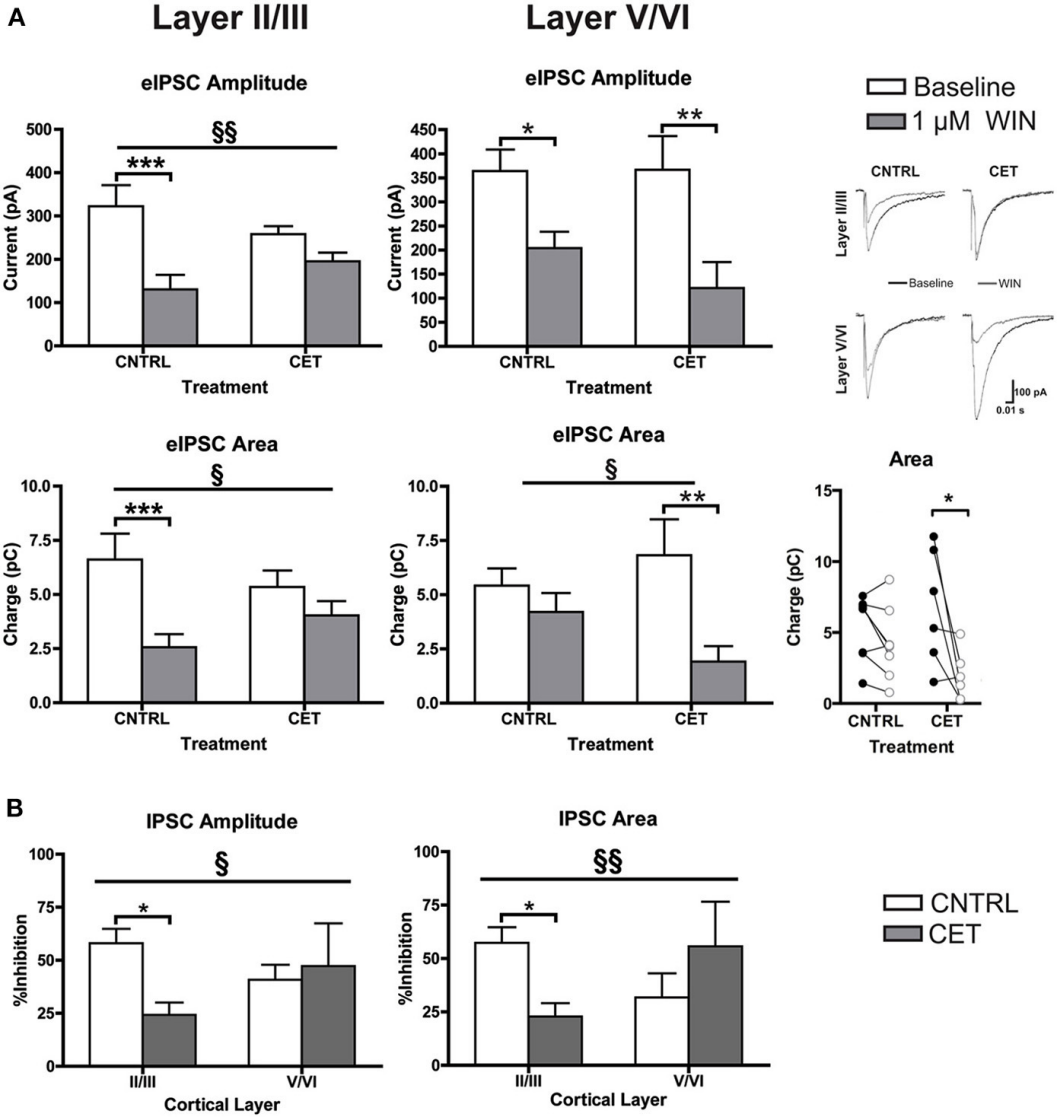
**CB1-mediated inhibition of GABAergic synapses on layer II/III PNs is blunted following CET**. **(A)** The effect of WIN on evoked GABAA IPSCs recorded from layer II/III and layer V/VI PNs in control and CET cultures (*N* = 6–13). White bars represent baseline measures and gray bars represent IPSCs recorded following a 10 min application of WIN (1 μM). The traces to the right are representative examples from each of the four groups. Traces in black are baseline, and the superimposed gray traces are from the same neurons in the presence of WIN. **(B)** The percent inhibition of IPSCs by WIN in layer II/III and layer V/VI neurons from CET and control cultures. White bars represent control treated cultures and gray bars represent data from CET cultures. Right panel shows eIPSC area for individual neurons before (closed circles) and after (open circles) exposure to WIN. All data shown are mean ± s.e.m. Significant interactions are denoted by section signs (§) and significant *post-hoc* tests are indicated by asterisks (^*^). For all symbols conferring statistical significance: single symbol *p* < 0.05, double symbol *p* < 0.01, triple symbol *p* < 0.001.

Although levels of CB1 protein were not affected, withdrawal from CET produced a deficit in WIN's ability to enhance up-state amplitude suggesting a functional down-regulation of CB1 signaling. CB1 receptors are expressed throughout the cortical mantle, but results from our previous study showed that CB1-mediated depression of GABAergic synaptic transmission is more robust at layer II/III synapses compared to layer V/VI (Pava et al., 2014). To test whether CET affects CB1 signaling in a layer-specific fashion, the effects of WIN on evoked GABA IPSCs were determined for layer II/III and layer V/VI PNs in control and CET cultures. As shown in Figure 3A, there was a significant interaction between treatment and response to WIN for measure of eIPSC amplitude [Two-Way repeated measures ANOVA, *F*_(1, 23)_ = 7.96, *p* = 0.0097] and area [*F*_(1, 23)_ = 5.36, *p* = 0.030] in layer II/III PNs. *Post-hoc* tests revealed that WIN reduced eIPSC amplitude [Bonferroni adjusted *t*-test, *t*_(23)_ = 5.82, *p* < 0.0001] and area [*t*_(23)_ = 4.76, *p* = 0.0002] in control cultures but not in CET cultures [amplitude: *t*_(23)_ = 1.99, *p* = 0.12; area: *t*_(23)_ = 1.62, *p* = 0.24] indicating that CET produced a functional down-regulation of CB1 signaling at GABAergic terminals onto layer II/III PNs. In layer V/VI PNs, there was a main effect of WIN to reduce eIPSC amplitude [Two-Way repeated measures ANOVA, *F*_(1, 12)_ = 19.18, *p* = 0.0009] that was driven by reductions in IPSCs from both control [Bonferroni adjusted *t*-test, *t*_(12)_ = 2.64, *p* = 0.043] and CET [*t*_(23)_ = 3.50, *p* = 0.0087] cultures. Measures of eIPSC area in layer V/VI neurons also displayed a WIN × Treatment interaction [Two-Way repeated measures ANOVA, *F*_(1, 12)_ = 4.78, *p* = 0.049], however, unlike results from layer II/III PNs, IPSCs in CET cultures were significantly inhibited by WIN (Bonferroni adjusted *t*-test, *t*_(23)_ = 5.82, *t*_(12)_ = 3.85, *p* = 0.0046]. Although WIN inhibited IPSC area in 6 out of 8 neurons from control cultures (Figure 3, inset), as a group, this inhibition was not statistically significant. [*t*_(12)_ = 1.10, *p* = 0.59].

To compare the effects of CET on WIN-induced inhibition of GABA_A_ IPSCs between cortical layers, the percent inhibition of eIPSCs was calculated (Figure 3B). There was a significant interaction between cortical layer and treatment for measures of IPSC amplitude (Two-Way ANOVA, *F*_(1, 37)_ = 4.50, *p* = 0.041]. The interaction was driven by a significant reduction in WIN-induced inhibition of IPSCs following CET that was selective for layer II/III PNs [Bonferroni adjusted *t*-test, *t*_(37)_ = 2.86, *p* = 0.014] and not layer V/VI PNs [*t*_(37)_ = 0.43, *p* > 0.99]. A similar effect was observed for measures of eIPSC area where there was an interaction between treatment and cortical layer [Two-Way ANOVA, *F*_(1, 36)_ = 7.88, *p* = 0.0080]. Again the inhibition by WIN of eIPSCs on layer II/III PNs was blunted by CET [Bonferroni adjusted *t*-test, *t*_(36)_ = 2.74, *p* = 0.019], and there was no difference in the response to WIN for eIPSCs recorded from layer V/VI cells [*t*_(36)_ = 1.45, *p* = 0.31]. These data demonstrate that there is a functional down-regulation of CB1 at GABAergic synapses on layer II/III PNs.

**Figure 4:**
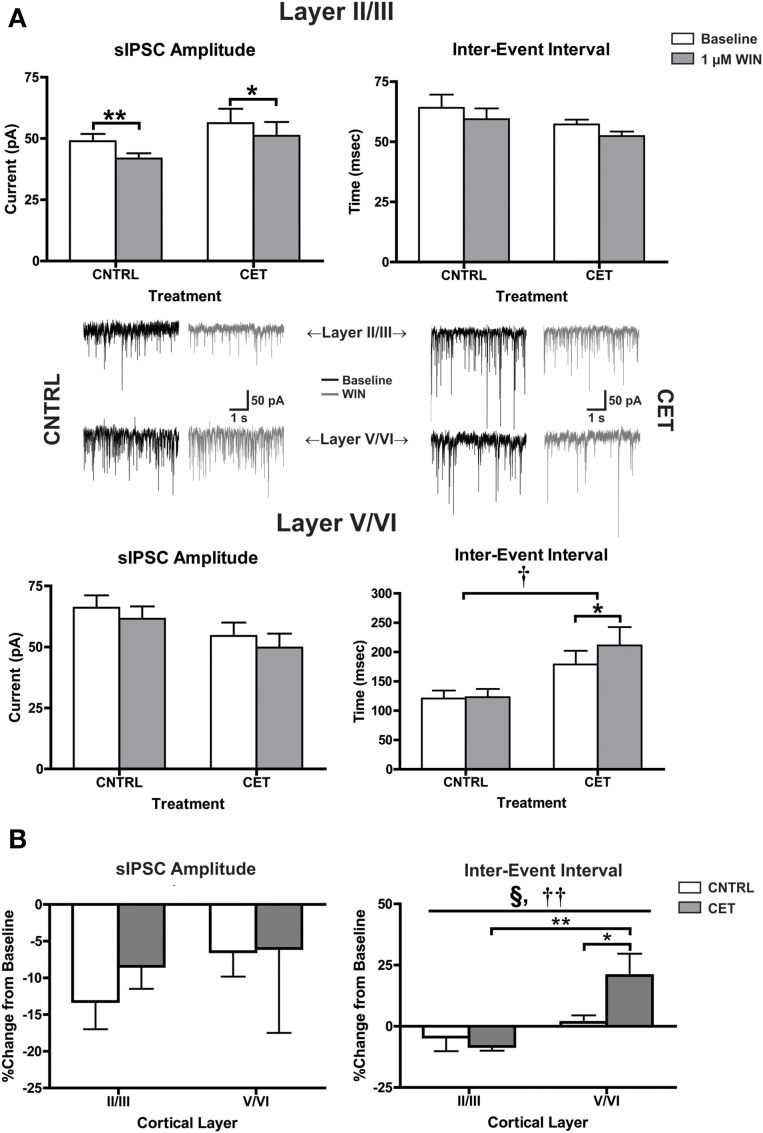
**CB1-mediated inhibition of GABAergic sIPSCs onto layer V/VI PNs is enhanced following CET**. **(A)** The effect of WIN on spontaneous GABAA IPSCs recorded from layer II/III and layer V/VI PNs in control and CET cultures (*N* = 7–12). White bars represent baseline measures and gray bars represent IPSCs recorded following a 10 min application of WIN (1 μM). Traces in the middle of **(A)** are representative examples from each of the four groups. The group of traces to the right is from control cultures, and traces to the left are from CET cultures. The top row of traces is from layer II/III PNs, and the bottom row is from layer V/VI. Black traces represent baseline recordings, and the adjacent gray traces are from the same neuron after a 10 min WIN application. All data shown are mean ± s.e.m. Symbols: †, main effect of EtOH treatment; ^*^, significant pair-wise comparison. **(B)** the percent change of sIPSCs from layer II/III and layer V/VI neurons in control and CET cultures. All data shown are mean ± s.e.m. Symbols: §, interaction; †, main effect of cortical layer; ^*^, significant *post-hoc* comparisons. For all symbols conferring statistical significance: single symbol *p* < 0.05, double symbol *p* < 0.01.

In the experiments with eIPSCs, the stimulus intensity was adjusted for each cell to obtain an IPSC amplitude of approximately 300 pA. Because the intensity of the synaptic response was experimenter defined, it is not possible to determine whether CET alters basal IPSC parameters. To address this concern, recordings of spontaneous IPSCs (sIPSCs) were obtained before and after applying WIN (1 μM) to layer II/III and layer V/VI neurons in CET and control cultures (Figure 4A). In layer II/III PNs, there was a significant main effect of WIN to reduce sIPSC amplitude [Two-Way repeated measures ANOVA, *F*_(1, 20)_ = 20.91, *p* = 0.0002] that was due to reductions in sIPSC amplitude in both control [Bonferroni adjusted *t*-test, *t*_(20)_ = 3.76, *p* = 0.0025] and CET cultures [*t*_(20)_ = 2.71, *p* = 0.027]. In layer II/III PNs, neither WIN [Two-Way repeated measures ANOVA, *F*_(1, 21)_ = 3.84 *p* = 0.064] nor CET [*F*_(1, 21)_ = 1.91, *p* = 0.18] altered the inter-event interval of sIPSCs. However, in layer V/VI PNs, there was a main effect of CET to increase the IEI of sIPSCs [Two-Way repeated measures ANOVA, *F*_(1, 13)_ = 7.38, *p* = 0.018] suggesting a down-regulation in basal GABAergic signaling in this layer following CET. In addition, there was also a significant main effect of WIN [*F*_(1, 13)_ = 5.49, *p* = 0.036] to increase the IEI of sIPSCs on layer V/VI PNs in CET cultures [Bonferroni adjusted *t*-test, *t*_(13)_ = 2.87, *p* = 0.027] but not controls [*t*_(13)_ = 0.19, *p* > 0.99]. There was no effect of either CET [Two-Way repeated measures ANOVA, *F*_(1, 15)_ = 2.62, *p* = 0.13] or WIN [*F*_(1, 15)_ = 3.22, *p* = 0.093] on sIPSC amplitude on layer V/VI PNs.

To directly compare changes in CB1-mediated signaling between cortical layers, the percent change of sIPSC amplitude and IEI were calculated (Figure 4B). There was no effect of either cortical layer [Two-Way ANOVA, *F*_(1, 36)_ = 0.78, *p* = 0.38] or CET [*F*_(1, 36)_ = 0.25, *p* = 0.62] on CB1-mediated inhibition of sIPSC amplitude. However, there was significant interaction between cortical layer and treatment [Two-Way ANOVA, *F*_(1, 35)_ = 5.00, *p* = 0.032] and a main effect of cortical layer [*F*_(1, 35)_ = 11.53, *p* = 0.0017] on the IEI of sIPSCs. This interaction was due to a WIN-induced increase in the IEI observed in layer V/VI PNs from CET cultures that was not observed in layer V/VI PNs from control cultures [Bonferroni adjusted *t*-test, *t*_(35)_ = 2.42, *p* = 0.042] or layer II/III PNs from CET cultures [*t*_(35)_ = 3.81, *p* = 0.0011]. These data provide further support for a gain of function for CB1 signaling at GABAergic synapses onto layer V/VI synapses following CET.

### CET does not alter CB1 signaling at glutamatergic synapses

**Figure 5:**
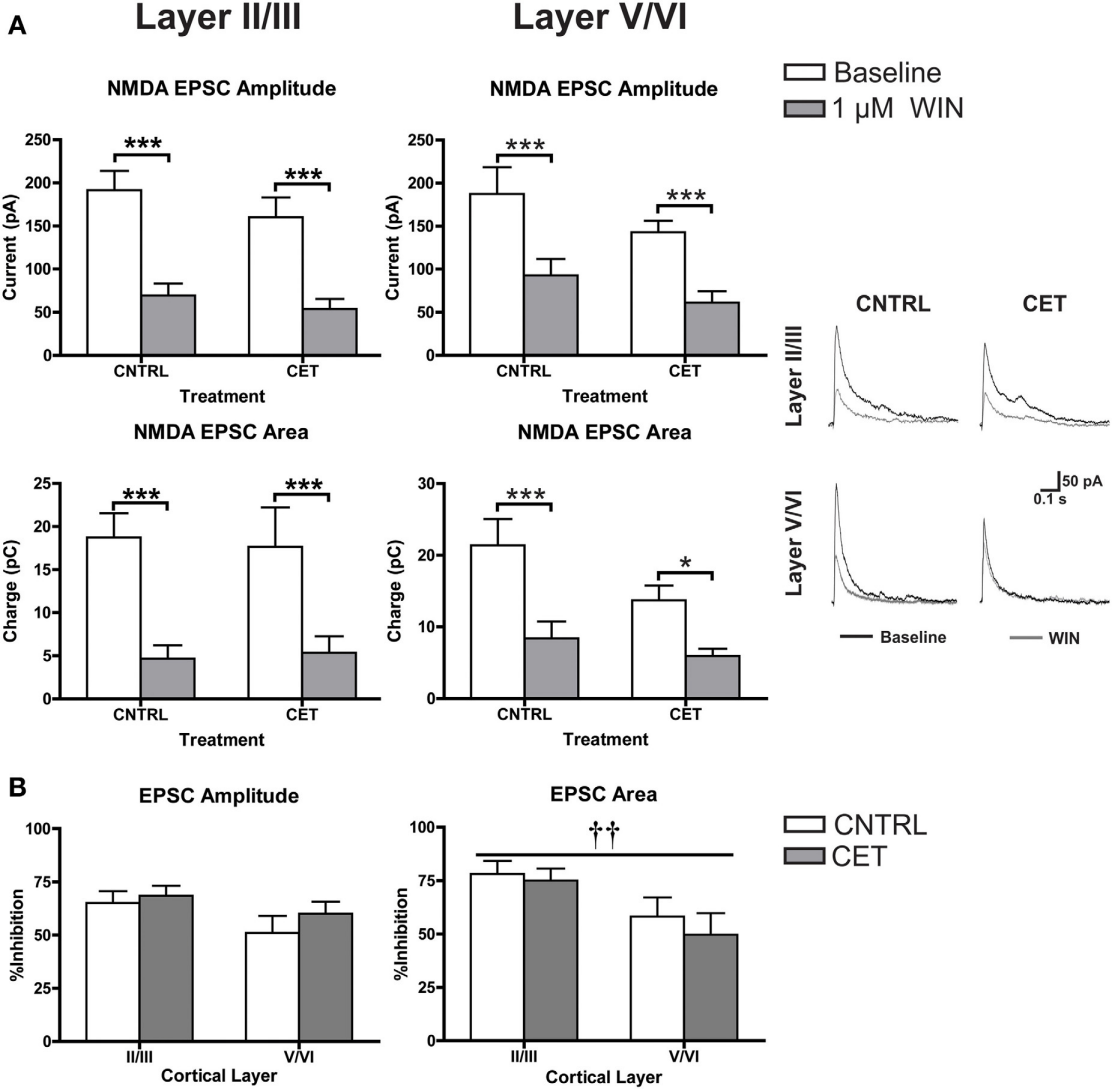
**CB1-mediated inhibition of glutamatergic transmission is not altered by CET**. **(A)** The effect of WIN on evoked NMDA EPSCs recorded from layer II/III and layer V/VI PNs in control and CET cultures (*N* = 8–9). White bars represent baseline measures and gray bars represent EPSCs recorded following a 10 min application of WIN (1 μM). The traces to the right are representative examples from each of the four groups. Traces in black are baseline, and the superimposed gray traces are from the same neurons in the presence of WIN. **(B)** The percent inhibition of EPSCs by WIN in layer II/III and layer V/VI neurons from CET and control cultures. White bars represent control treated cultures and gray bars represent data from CET cultures. All data shown are mean ± s.e.m. Symbols: †, main effect of cortical layer; ^*^, significant pair-wise comparison. For all symbols conferring statistical significance: single symbol *p* < 0.05, double symbol *p* < 0.01, triple symbol *p* < 0.001.

In addition to down-regulating CB1 at GABAergic terminals, the blunted response of up-states in CET cultures to WIN could be due to an up-regulation of CB1 at glutamate synapses that helps to balance the disinhibition caused by activation of CB1 at GABAergic terminals. At least one study has demonstrated increased CB1 expression in the PFC of alcohol exposed rodents suggesting this may be possible (Rimondini et al., 2002). To explore this idea in the current study, NMDA EPSCs were recorded from layer II/III and layer V/VI PNs in CET and control cultures before and after the application of WIN (1 μM; Figure 5A). In layer II/III PNs, there was no effect of CET on EPSC amplitude [Two-Way repeated measures ANOVA, *F*_(1, 15)_ = 0.99, *p* = 0.34] or area [*F*_(1, 15)_ = 0.0031, *p* = 0.96], but there was a main effect of WIN to reduce both EPSC amplitude [*F*_(1, 15)_ = 106.8, *p* < 0.0001] and area [*F*_(1, 15)_ = 55.59, *p* < 0.0001]. WIN reduced EPSC amplitude in both control [Bonferroni adjusted *t*-test, *t*_(15)_ = 8.053 *p* < 0.0001] and CET cultures [*t*_(15)_ = 6.61, *p* < 0.0001], and produced a similar reduction in EPSC area in control [*t*_(15)_ = 5.80, *p* < 0.0001] and CET [*t*_(15)_ = 4.78, *p* = 0.0005] cultures. These data indicate that treatment with EtOH did not alter the sensitivity of glutamate terminals on layer II/III neurons to WIN. Similar to the findings in layer II/III neurons, there was no effect of CET on NMDA EPSCs recorded from layer V/VI PNs for measures of either amplitude [Two-Way repeated measures ANOVAs, *F*_(1, 17)_ = 2.12, *p* = 0.16] or area [*F*_(1, 16)_ = 3.18, *p* = 0.093], but there was a main effect of WIN to reduce EPSC amplitude [*F*_(1, 17)_ = 69, *p* < 0.0001] and area [*F*_(1, 16)_ = 26.13, *p* = 0.0001]. Pair-wise comparisons found that WIN application significantly reduced EPSC amplitude in both treatment groups [Bonferroni adjusted *t*-test, control: *t*_(17)_ = 5.58, CET: *t*_(17)_ = 6.16, *p* < 0.0001], and similarly, WIN reduced EPSC area in both control [*t*_(16)_ = 4.52, *p* = 0.0007] and CET cultures [*t*_(16)_ = 2.71, *p* = 0.031]. The percent inhibition of EPSCs by WIN was calculated and these values were compared across cortical layers (Figure 5B). CET did not affect the sensitivity of EPSC amplitude to inhibition by WIN [Two-Way ANOVA, *F*_(1, 32)_ = 1.01, *p* = 0.32], and there was no difference in the amplitude of evoked NMDA EPSCs between cortical layers [*F*_(1, 32)_ = 3.27, *p* = 0.080]. However, measurements of EPSC area indicate that currents recorded from layer II/III were inhibited to a greater extent that those recorded from layer V/VI [main effect of cortical layer, two-way ANOVA, *F*_(1, 32)_ = 7.51, *p* = 0.0099], but again there was no effect of CET [*F*_(1, 32)_ = 0.51, *p* = 0.48]. These results show that CET does not alter CB1 signaling at glutamatergic synapses onto layer II/III or layer V/VI pyramidal neurons.

## Discussion

**Figure 6:**
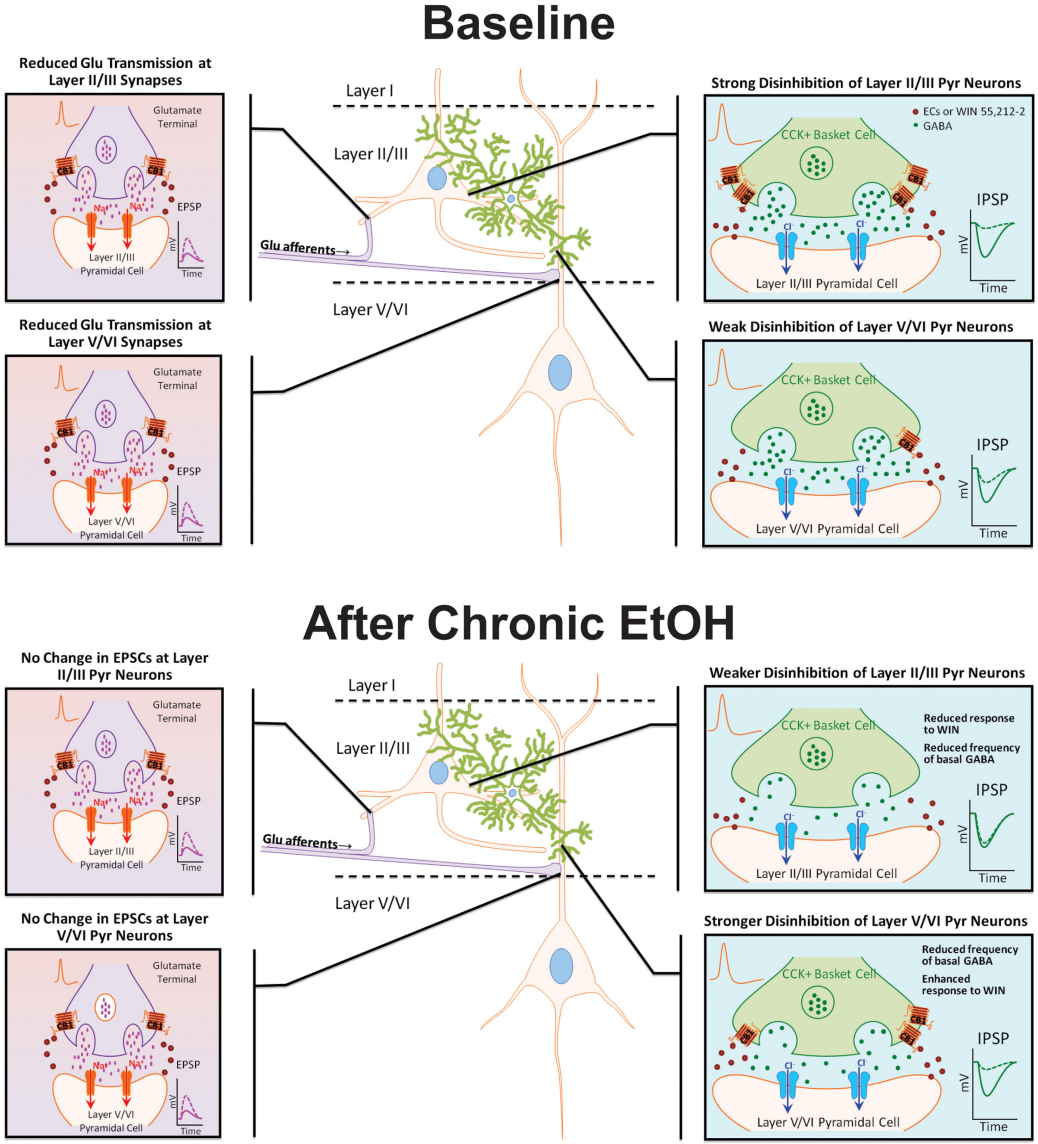
**Model diagram summarizing the effects of CB1 activation on specific synapses under basal conditions and following chronic EtOH treatment**. Under baseline conditions, CB1 activation reduces GABAergic and NMDA receptor-mediated glutamate transmission at synapses on layer II/III and layer V/VI PNs. The inhibition of GABAergic transmission onto layer II/III PNs is much greater than at GABA synapses on layer V/VI neurons. There is no difference in the inhibition of glutamate transmission between layers. Following chronic EtOH treatment, the CB1-mediated disinhibition of layer II/III PNs is reduced, and there is a gain of function in CB1 signaling at deep-layer GABA synapses effectively switching the cannabinoid sensitivity between cortical layers. Chronic EtOH treatment does not affect CB1 signaling at glutamate synapses. Abbreviations: CB1, cannabinoid receptor 1; CCK+, cholecystokinin positive; Cl−, chloride ion, ECs, endocannabinoids; EPSP, excitatory post-synaptic potential; GABA, γ-amino-butyric acid; IPSP, inhibitory post-synaptic potential; Na+, sodium ion; WIN, WIN 55,212-2.

Chronic exposure to EtOH alters components of the EC system, but few studies have examined the effect of EtOH on cannabinoid receptor expression and function in the PFC. In the present investigation, organotypic cultures of mouse PFC were chronically exposed to EtOH for 10 days and subsequently allowed to withdraw for 4 days to examine persistent effects on network activity. Reminiscent of findings from CB1 KO cultures (Pava et al., 2014), up-state duration was significantly enhanced following withdrawal from EtOH treatment, and despite no significant change in CB1 protein expression, the response of up-states to WIN was blunted at this time point. As the response of PSCs to WIN is dependent on cortical layer and PSC type (i.e., glutamatergic or GABAergic; Pava et al., 2014), experiments were performed to determine if the reduction in CB1 signaling after CET showed any layer selectivity (summarized in Figure 6). CET significantly reduced the CB1-mediated inhibition of layer II/III GABAergic transmission, and there was an increase in CB1-mediated inhibition of GABA IPSCs on layer V/VI neurons that was evident in some measures but not others. Furthermore, CET did not alter the sensitivity of NMDA EPSCs to WIN indicating that the effects of CET on the up-state response to WIN are likely due to altered CB1 signaling at GABAergic synapses. A caveat to this conclusion is that, in this study, we did not examine a role for CB2 receptors that are also activated by WIN and have been suggested to have actions on cortical activity (Ortega-Alvaro et al., 2011). However, as our previous findings show that the actions of WIN on up-state parameters are eliminated in PFC slice cultures prepared from CB1 knockout mice (Pava et al., 2014), we feel that invoking a CB2 mediated mechanism for any of the observed effects is not warranted.

### Network activity and CB1 expression and function are disrupted following withdrawal from CET

Alcohol dependent patients display poor performance on PFC-dependent tasks and this is correlated with reduced power of delta frequency EEG measurements of frontal cortical function (Kamarajan et al., 2004; Chen et al., 2007). In addition, the sleep efficiency and time spent in slow-wave sleep are reduced in rodent models of dependence (Veatch, 2006). Up-states are the cellular correlate of the slow-oscillation that predominates during deep-stage non-REM sleep (Amzica and Steriade, 1998) and can be reconstituted *in vitro* using organotypic neocortical cultures that are amenable to studies using highly controlled chronic drug treatment (Seamans et al., 2003). In the present work, up-state duration was longer in cultures exposed to CET suggesting that EtOH treatment and withdrawal leads to neuroadaptive changes that contribute to altered network dynamics.

In our previous study, cultures with a chronic deficit of CB1 receptors (i.e., cultures prepared from CB1 KO mice) also displayed enhanced duration of up-states as compared to *wt* cultures (Pava et al., 2014), suggesting a role for the EC system in regulating cortical network activity. Studies in the hippocampus have reported a down-regulation of CB1 following the induction of EtOH tolerance/dependence that rebounds above control levels during withdrawal (Vinod et al., 2006; Mitrirattanakul et al., 2007), and a study in the cortex found increased CB1 gene expression following a 3 week withdrawal from chronic ethanol administration (Rimondini et al., 2002). It is important to note that both of these investigations used *in vivo* regimens of ethanol treatment and withdrawal that lasted many weeks. In the present study, ethanol exposure and withdrawal was more limited due to the shorter lifespan of the *in vitro* cortical cultures. Consequently, the data in the present study are consistent with most findings in neocortical and hippocampal tissue in rodent models of dependence, although they differ somewhat from those of Vinod et al. (2006). The decrease in CB1 binding and coupling in the cortex observed by Vinod *et al* occurred immediately after removal of mice from 72 h of continuous treatment with EtOH vapor, while in the present study, CB1 protein expression in CET cultures were similar to control levels immediately following treatment. Furthermore, Vinod et al. (2006) report that CB1 binding and G-protein coupling returned to control levels following a 24 h withdrawal, but in the present work, CB1 protein expression was unchanged relative to controls following a 4 day withdrawal from CET despite a reduction in WIN-induced alteration of up-state amplitude. These results suggest that EtOH may induce differential effects on CB1 protein expression, G-protein coupling efficiency, and perhaps cell surface expression that vary across both brain regions and EtOH exposure patterns. Future studies are needed to fully characterize the timecourse of fluctuations in CB1 expression and function over the course of EtOH treatment and withdrawal.

The mechanism behind EtOH's modulation of CB1 is likely to be due to alterations in the tone of EC transmitters in the brain. Previous studies reporting a decrease in CB1 receptors following EtOH treatment in rodents have found a correlated increase in tissue levels of AEA (Vinod et al., 2006) and 2-AG (Mitrirattanakul et al., 2007). Along these lines, alcohol preferring rats exhibit reduced FAAH activity in the PFC that is correlated with decreased CB1 function, and the alcohol preferring phenotype can be reconstituted in wistar rats by microinjecting a FAAH inhibitor into the PFC (Hansson et al., 2007). Similarly, FAAH KO mice exhibit enhanced EtOH preference compared to *wt* mice and have reduced CB1 binding in the CA1 region of the hippocampus (Vinod et al., 2008). These results demonstrate that either pharmacologically induced EtOH dependence or a genetic predisposition to consume EtOH is correlated with enhanced AEA tone and decreased CB1 expression. However, studies using chronic AEA administration in FAAH KO mice or chronic administration of FAAH inhibitors to *wt* mice consistently find no change in CB1 expression or agonist-stimulated GTPγS binding (Falenski et al., 2010; Schlosburg et al., 2010, 2014). In contrast, these studies do observe reduced CB1 expression and G-protein coupling with chronic up-regulation of 2-AG following MAGL inhibition. Results reported in Pava et al. (2014) suggest that EC tone is permissive for the generation of up-states in the neocortex. As a consequence, it is possible that the alteration in CB1 function observed in the present study is due to an augmentation of EC tone, and future work should be directed at determining the effect of chronic EtOH on EC tone and FAAH and MAGL activity in the PFC.

### Reduced sensitivity to WIN in CET cultures is due to a reduction in CB1 signaling at GABAergic synapses on layer II/III PNs

As reported previously, the modulation of up-state parameters by WIN was associated with a selective disinhibition of layer II/III PNs (Pava et al., 2014). The results from this study complement these findings by extending the work to include data from cultures that have undergone withdrawal from chronic EtOH. In humans, withdrawal from chronic EtOH is associated with a syndrome of enhanced CNS excitability (Saitz, 1998), and similar observations have been made in rodent models where repeated withdrawals from chronic EtOH enhance electrographic seizures and handling-induced convulsions (Veatch and Becker, 2002). In this study, CET displayed a blunted network response to WIN and this was accompanied by a lack of sensitivity to the effects of WIN on evoked GABAergic IPSCs onto layer II/III PNs.

Layer II/III PNs receive the majority of their inputs from distal regions of cortex, and in addition, they have a high degree of intra-laminar connectivity compared to deep-layer PNs (Douglas and Martin, 2004). A CB1-mediated reduction of GABAergic input in layer II/III PNs is predicted to facilitate recruitment of neurons that receive intra-laminar connections as well as laver V/VI PNs that receive inputs from layer II/III. As a consequence, the effects of CB1 activation at layer II/III GABA terminals may synergize with the enhanced glutamatergic and reduced GABAergic drive following CET that predisposes the cortical network to increased excitability. Although measures of network activity immediately following the removal of EtOH were not made in this study, hippocampal explants demonstrate seizure-like activity after the immediate cessation of long-term EtOH treatment that is due to enhanced NMDA receptor activation (Hendricson et al., 2007). If similar conditions exist in the PFC, then increased EC release would be expected, and reduced CB1 function at layer II/III GABAergic synapses may serve as a compensatory mechanism to reduce activity in the network.

In contrast to the effects observed in layer II/III, the sensitivity of eIPSCs in layer V/VI neurons to WIN was enhanced following CET, indicating that the laminar distribution of CB1 function at GABAergic synapses is reversed in CET cultures with respect to controls. This result was surprising given the overall decreased sensitivity of up-states to WIN following CET. Based upon the present data, it is not clear if this enhancement represents up-regulation of CB1 function, but if so, it seems likely that the reduced function observed in layer II/III is overcomes this effect with respect to network dynamics.

Layer V/VI PNs represent the major projection cells of the cortex, and disinhibition of these neurons by cannabinoids would increase the gain of cortical output. One consequence of increased disinhibition of layer V/VI PNs would be to enhance excitatory drive in the cultures. CET cultures were found to display longer up-states than controls, and this disinhibitory mechanism may prolong up-state duration. However, CB1 KO cultures also display prolonged up-states, so the functional consequences of up-regulating the CB1-mediated disinhibition of layer V/VI PNs are difficult to determine with the present dataset. Additionally, the predicted effects of enhancing the disinhibition of deep-layer PNs stand in contrast to studies suggesting decreased PFC function in addiction (Goldstein et al., 2004; Kamarajan et al., 2004; Volkow et al., 2004). It is possible that the alteration to CB1 function may be in response to a reduction in EC tone due to lower cellular activity, and along these lines, reduced EC tone is predicted to facilitate an up-regulation of CB1 as the application of CB1 antagonists is known to increase trafficking of this receptor to the plasma membrane in cultured neurons (Leterrier et al., 2006; Howlett et al., 2011). Consequently, the changes in CB1-signaling in layer V/VI may arise from allostatic processes characteristic of addiction (Koob and Le Moal, 2001).

A competing mechanism that could reduce sensitivity of up-states to WIN would be an enhancement of CB1 function on glutamatergic terminals thus reducing overall excitation. However, WIN was equally effective in reducing EPSCs in EtOH treated and control cultures suggesting that sensitivity was unaffected by ethanol treatment. It is possible that at the concentration of WIN (1 μM) used for these experiments there was a ceiling effect for NMDA EPSCs that obscured an ethanol-induced change in cannabinoid sensitivity. However, this seems unlikely since this same concentration of WIN was also used in the studies recording up-states, and if altered CB1 signaling at glutamate synapses were responsible for the blunted effect on up-states then this should be apparent in the recordings of NMDA EPSCs. Therefore, it is unlikely that an increased sensitivity of excitatory synapses to WIN mediates the EtOH-induced changes in the cortical network response to CB1 activation.

The findings from this study complement and extend those from our previous report by demonstrating that chronic ethanol exposure results in altered network activity in the PFC that is associated with specific changes in the laminar distribution of CB1 signaling (Figure 6). These changes in the EC system likely result from the increased excitability of the cortex that emerges during withdrawal from CET, and they have potential ramifications for cortical network activity. After the CNS excitability observed during the acute phase of withdrawal subsides, alcohol dependent patients often display cognitive deficits that are indicative of reduced function of the prefrontal cortices. Decreased cortical control of behaviors associated with drug-seeking is a hallmark of addiction, and this state can persist into abstinence raising the likelihood of relapse. Consequently, future studies in intact systems should be conducted to address whether the synapse-specific alterations in CB1 signaling observed here contribute to reduced cortical function associated with alcohol dependence.

### Conflict of interest statement

The authors declare that the research was conducted in the absence of any commercial or financial relationships that could be construed as a potential conflict of interest.
